# 

**Published:** 1879-04

**Authors:** 


### Table of Contents.

### ORIGINAL COMMUNICATIONS.

A CASE OF OBSTETRICS. *By E. A. Cobleigh, M.D., Athens, Tenn.*.. 1

A NEW UTERINE DILATOR. *By Eli Griffin, M.D., Atlanta, Ga*...... 5

### REPORTS OF SOCIETIES.

Atlanta Academy of Medicine.... 7
Toledo Medical Association.... 14
Proceedings of the Suffolk District Medical Society.... 15

### SELECTIONS.

Partial Retention of the Placenta after Labor.... 21
Dr. C. W. Long, the Discoverer of Anæsthesia.... 27
Abortive Treatment of Bubo.... 34
A Practical Diet Table for Diabetics.... 35
Sympathetic Insanity proceeding from the Rectum.... 37
On Aconite as a Therapeutical Agent.... 40

### EDITORIAL.

Tax on Doctors.... 43
In Memoriam.... 51
Congregation of Medical Men in Atlanta.... 51
Bulletin of the Public Health.... 54

### BIBLIOGRAPHICAL.

The Practitioner's Reference Book.... 46
Practical Gynæcology.... 46
Handbook of the Practice of Medicine.... 47
Clinical Lectures on Diseases Peculiar to Women.... 47
Yellow Fever.... 48
A Clinical Treatise on Diseases of the Liver.... 48
The Diseases of Live Stock and their most Efficient Remedies.... 49
Pamphlets Received.... 49

### EXCERPTA.

Treatment of Alcoholism.... 56

Patent Medicines in Drug Stores.... 57

The Largest Infant on Record.... 58

The Metrical System in Great Britain.... 58

Treatment of Dyspepsia.... 59

Capsicum and Quinine.... 60

Counter Practice in France.... 60

Spanked.... 61

Cement for Wood and Iron.... 61

Diphtheria.... 62

Information Wanted.... 62

"Eye and Ear Infirmary," or "Oculist and Aurist.".... 63

Gurjun Balsam in Gonorrhœa.... 63

Cholera Infantum.... 63

Religion and Chloroform.... 64

Gonorrhœa.... 64

Removal of Steel Rust.... 64

